# Phenome-wide investigation of health outcomes associated with genetic predisposition to loneliness

**DOI:** 10.1093/hmg/ddz219

**Published:** 2019-09-13

**Authors:** Abdel Abdellaoui, Sandra Sanchez-Roige, Julia Sealock, Jorien L Treur, Jessica Dennis, Pierre Fontanillas, Sarah Elson, The 23andme Research Team, Michel G Nivard, Hill Fung Ip, Matthijs van der Zee, Bart M L Baselmans, Jouke Jan Hottenga, Gonneke Willemsen, Miriam Mosing, Yi Lu, Nancy L Pedersen, Damiaan Denys, Najaf Amin, Cornelia M van Duijn, Ingrid Szilagyi, Henning Tiemeier, Alexander Neumann, Karin J H Verweij, Stephanie Cacioppo, John T Cacioppo, Lea K Davis, Abraham A Palmer, Dorret I Boomsma

**Affiliations:** 1 Department of Psychiatry, Amsterdam UMC, University of Amsterdam, Amsterdam, The Netherlands; 2 Department of Biological Psychology, Vrije Universiteit, Amsterdam, The Netherlands; 3 Department of Psychiatry, University of California San Diego, La Jolla, CA, USA; 4 Vanderbilt Genetics Institute, Division of Genetic Medicine, Department of Medicine, Vanderbilt University, Nashville, TN, USA; 5 School of Experimental Psychology, University of Bristol, Bristol, UK; 6 MRC Integrative Epidemiology Unit, University of Bristol, Bristol, UK; 7 23andMe, Inc., Mountain View, CA, USA; 8 Department of Neuroscience, Karolinska Institutet, Stockholm, Sweden; 9 Department of Medical Epidemiology and Biostatistics, Karolinska Institutet, Stockholm, Sweden; 10 Genetic Epidemiology Unit, Department of Epidemiology, Erasmus Medical Center, Rotterdam, The Netherlands; 11 Translational Epidemiology, Faculty Science, Leiden University, Leiden, The Netherlands; 12 Department of Epidemiology, Erasmus Medical Center, Rotterdam, The Netherlands; 13 Department of Psychiatry, Erasmus Medical Center, Rotterdam, The Netherlands; 14 Department of Child and Adolescent Psychiatry/Psychology, Erasmus Medical Center, Rotterdam, The Netherlands; 15 Center for Cognitive and Social Neuroscience, Department of Psychology, The University of Chicago, Chicago, Illinois, USA

## Abstract

Humans are social animals that experience intense suffering when they perceive a lack of social connection. Modern societies are experiencing an epidemic of loneliness. Although the experience of loneliness is universally human, some people report experiencing greater loneliness than others. Loneliness is more strongly associated with mortality than obesity, emphasizing the need to understand the nature of the relationship between loneliness and health. Although it is intuitive that circumstantial factors such as marital status and age influence loneliness, there is also compelling evidence of a genetic predisposition toward loneliness. To better understand the genetic architecture of loneliness and its relationship with associated outcomes, we extended the genome-wide association study meta-analysis of loneliness to 511 280 subjects, and detect 19 significant genetic variants from 16 loci, including four novel loci, as well as 58 significantly associated genes. We investigated the genetic overlap with a wide range of physical and mental health traits by computing genetic correlations and by building loneliness polygenic scores in an independent sample of 18 498 individuals with EHR data to conduct a PheWAS with. A genetic predisposition toward loneliness was associated with cardiovascular, psychiatric, and metabolic disorders and triglycerides and high-density lipoproteins. Mendelian randomization analyses showed evidence of a causal, increasing, the effect of both BMI and body fat on loneliness. Our results provide a framework for future studies of the genetic basis of loneliness and its relationship to mental and physical health.

## Introduction

Loneliness is a universal human experience that has been documented across cultures and generations. According to the evolutionary theory of loneliness (1), this painful feeling corresponds to an aversive response to a discrepancy between a people’s desired and perceived level of social connectedness (2,3). This definition emphasizes the desired level of social connection and highlights the difference between loneliness and solitude. Unlike solitude, the signal associated with loneliness has likely evolved to motivate humans and other social animals to seek and improve the salutary social connections needed to help them survive and reproduce (4). Loneliness serves as an emotional warning or signal that there is an emotional imbalance in one’s social network, regardless of the size of that network. Feeling lonely is also very common; about 5–30% of adults in Western populations report some degree of loneliness, while the actual prevalence may be higher since loneliness is stigmatized in many cultures (5–7).

Both social isolation and chronic high levels of loneliness are strongly correlated with negative health outcomes; chronic loneliness has a stronger association with early mortality than obesity does (8). A long-running longitudinal study on physical and mental health, the Harvard Study of Adult Development, has concluded that the warmth of one’s relationships has the greatest impact on wellbeing and life satisfaction (9). Findings like these suggest that loneliness is a public health concern. Although these studies demonstrate a clear and strong correlation between loneliness and increased morbidity and mortality, the causality and etiology of the relationship between loneliness and mental and physical health is unclear. For example, loneliness may cause poor health, or, alternatively, poor health may cause loneliness directly or indirectly by disrupting social networks.

Multiple factors influence variation in the experience of chronic loneliness (1). Most studies have focused on circumstantial factors such as marital status, age, and sex (10–13). However, there are also innate individual differences in the propensity to feel lonely. Heritability estimates based on twin and family data suggest that ~37% of the variation in loneliness levels is explained by genetic factors (14) and studies analyzing molecular genetic data estimated that the aggregate of common genetic variants accounted for 4–27% in individual differences in loneliness (15–17). A genome-wide association study (GWAS) of social interaction and isolation in the UK Biobank sample identified 15 common genetic variants associated with loneliness (17). Here, we extend the GWAS based meta-analysis for loneliness including more than half-a-million subjects of European descent from various cultural backgrounds. In addition to the UK biobank (UKB), we analyzed data from 23andMe (USA), the Health and Retirement Study (USA), the Netherlands Twin Register (NTR), and the Swedish Twin Registry (STR).

We performed a series of analyses on the new summary statistics (18) to further elucidate the biological basis underlying the propensity to feel lonely and the genetic overlap between loneliness and complex human traits related to personality, cognition, reproduction, substance use, social connections and physical and mental health. Next, we carried out a phenome-wide association study (PheWAS). PheWAS has emerged as a method to screen for associations between genetic measures and a range of phenotypes, such as those measured in electronic health records (EHR) (19, 20). For many phenotypes, EHR may provide more objective measures of physical and mental health than self-reported health data, which may not be readily known by patients (e.g. lab values) or can be distorted by mood and recall bias. Since the time of their original publications, the PheWAS approaches expanded beyond the analysis of a single SNP to include analysis of polygenic risk scores (21). We constructed a polygenic score of loneliness based on the estimated SNP effects from our new GWAS meta-analysis and performed a PheWAS in the Vanderbilt University Medical Center (VUMC) EHR and associated biobank by testing the association between the genetic risk for loneliness and 897 disease phenotypes and three types of clinically measured lipid levels. However, these analyses do not distinguish causal effects from pleiotropic effects. Therefore, we further tested for bidirectional causal relationships between loneliness and a selection of genetically correlated phenotypes by Mendelian randomization (MR) analysis. We performed a comprehensive characterization of the polygenic contribution to loneliness and extended this understanding to elucidate the genetic relationships between loneliness and health.

## Results

### GWAS meta-analysis

GWASs were run in seven cohorts with a total of 511 280 adult subjects, on dichotomous measures for loneliness for the largest cohort (UKB; 81 011 cases and 367 945 controls) and continuous measures for the other six cohorts (see Material and Methods for more details on the meta-analysis approach). The six continuous GWASs were first meta-analyzed separately and were then combined with the categorical GWAS in a meta-analysis using sample size-based weights (22). The proportion of phenotypic variance accounted for by all genotyped variants (SNP heritability) of the categorical loneliness measure in UKB and continuous loneliness measure were 8.1% (SE = 0.07) and 4.2% (SE = 0.07) respectively (see Supplementary Table 3). The genetic correlation between the categorical GWAS and the continuous GWAS (computed with LD-score regression) was.69 (*P* = 8.6 × 10^−16^). This non-unity genetic correlation may reflect the heterogeneity of the trait and/or measures, which has also been observed between different cohorts for other GWAS meta-analyses, such as major depressive disorder (MDD) (23). The SNP heritability in the meta-analysis was 6.6% (SE = 0.03), which accounts for approximately one fifth to one-quarter of the total heritability as estimated in twin-family studies. The estimate is ~2.4% higher than the SNP heritability estimate reported in an earlier GWAS on social interaction and social isolation in the UKB sample only (17).

**Figure 1:**
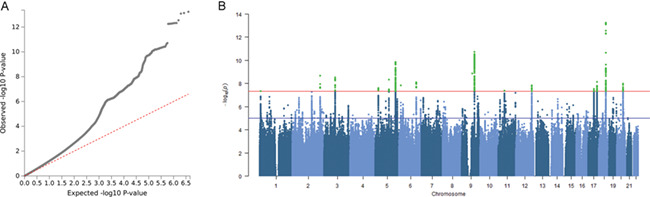
QQ-plot and Manhattan plot of meta-analysis on loneliness. (**A**) The QQ-plot shows a considerable inflation of association statistics (λ = 1.28), which is mostly due to true polygenic signal rather than population stratification (LD-score regression intercept = 0.99). (**B**) Manhattan Plot of the Loneliness GWAS meta-analysis showing 19 independent genome-wide significant associations from 16 loci.

The genomic inflation factor *λ* was 1.28 for the full meta-analysis (Fig. 1) and results from linkage disequilibrium score regression (LDSC) analysis (24) showed that this inflation was due to true polygenic signal (LDSC intercept = 0.99). We identified 19 independent genome-wide significant variants (*r*^2^ < 0.1), which were located in 16 genomic regions (i.e. within 250 kb; Fig. 1, Table 1, and Supplementary File 1). Twelve loci were within regions that were reported as significant in an earlier GWAS study on social interaction and social isolation in UKB alone (17), while four loci were novel (Table 1).

**Table 1:** 19 Independent genome-wide significant SNPs from 16 loci, with independence based on an *r*^2^ threshold 1, belonging to the same locus if they are within 250 kb (see Supplementary File 1 for more details on the significant SNPs)

**SNPs**	**CHR**	**BP (hg19)**	**A1/A2**	**STAND. BETA (SE)**	***P*-value**	**Gene**	**Position**	**Sign. in UKB only** ^**17**^
rs599550	18	53 252 388	A/G	−0.03 (.004)	5.88E–14	TCF4	Intronic	Yes
rs72627233	18	53 486 724	G/T	−0.02 (.004)	1.25E–08	RP11–397A16.3	Exonic	Yes
rs12458015	18	53 305 735	C/T	−0.02 (.003)	1.16E–09	TCF4	Intronic	Yes
rs13291079	9	96 360 650	C/T	0.02 (.003)	1.95E–11	PHF2	Intronic	Yes
rs4958586	5	152 248 567	A/G	−0.02 (0.003)	1.50E–10	AC091969.1	Intronic	Yes
rs773020	9	77 768 122	A/G	−0.03 (0.005)	1.43E–09	–	Intergenic	Yes
rs74338595	2	212 749 786	C/T	0.02 (0.003)	2.19E–09	ERBB4	Intronic	Yes
rs7626596	3	82 000 680	A/G	0.02 (0.003)	3.12E–09	–	Intergenic	Yes
rs171697	5	103 956 516	C/G	−.02 (0.003)	4.84E–09	RP11–6 N13.1	Intronic	Yes
rs11867618	17	65 875 587	A/G	−0.02 (0.004)	7.37E–09	BPTF	Intronic	
rs7209581	17	66 174 416	C/G	−0.02 (0.003)	4.76E–08	BPTF	Intergenic	Yes
rs7770860	6	131 186 393	C/T	−0.02 (0.003)	8.53E–09	EPB41L2	Intronic	Yes
rs348258	20	47 768 988	C/T	−0.02 (0.003)	1.06E–08	STAU1	Intronic	Yes
rs10456089	6	11 959 836	A/G	0.04 (0.006)	1.54E-08	–	Intergenic	Yes
rs11068917	12	118 791 120	A/C	−0.02 (0.004)	1.54E–08	TAOK3	Intronic	No
rs62347916	5	24 239 998	A/G	0.02 (0.003)	2.71E–08	–	Intergenic	No
rs2732650	17	44 344 988	C/G	−0.02 (0.004)	3.18E–08	RP11–259G18.1	Intronic	No
rs11039265	11	47 523 214	A/C	0.02 (0.003)	4.57E–08	CELF1	Intronic	Yes
rs159960	1	8 476 428	A/G	0.02 (0.003)	4.77E–08	RERE	Intronic	No

### MAGMA and S-PrediXcan gene-based analyses

To identify associations at a gene level, we performed two types of gene-based analyses using the GWAS meta-analysis association statistics: 1) MAGMA, which aggregates single-nucleotide polymorphism (SNP) effects at the gene level using positional annotations and 2) S-PrediXcan, which uses expression quantitative-trait loci annotations to assign SNPs to genes. The meta-analysis summary statistics formed the basis to compute gene-based *P*-values in MAGMA (25) and S-PrediXcan (26) for 18 714 and 13 037 protein-coding genes, respectively. In the MAGMA analysis, a total of 58 genes reached genome-wide significance at a Bonferroni corrected significance threshold of 2.67 × 10^−6^ (Supplementary Fig. 1). Seven of these genes (TCF4, PHF2, BPTF, STAU1, TAOK3, CELF1, RERE; Table 1) included at least one genome-wide significant SNP from the GWAS meta-analysis. Using S-PrediXcan (26), we identified 19 genes (of which 6 were also significant in the MAGMA analysis: C1QTNF4, MAPT, MST1, MTCH2, PLEKHM1 and SLC39A13) that were significantly associated with loneliness at a Bonferroni corrected significance threshold of *P* < 1.29 x 10^−6^ across 10 brain tissues: anterior cingulate cortex, caudate, cerebellum, cortex, cerebral hemisphere, hippocampus, hypothalamus, nucleus accumbens, prefrontal cortex and putamen (Supplementary Table 4).

### GWAS signals are significantly enriched for brain tissues and evolutionarily conserved regions

Next, we investigated if genetic effects on loneliness were enriched for loci with specific functional and tissue annotations.

**Figure 2:**
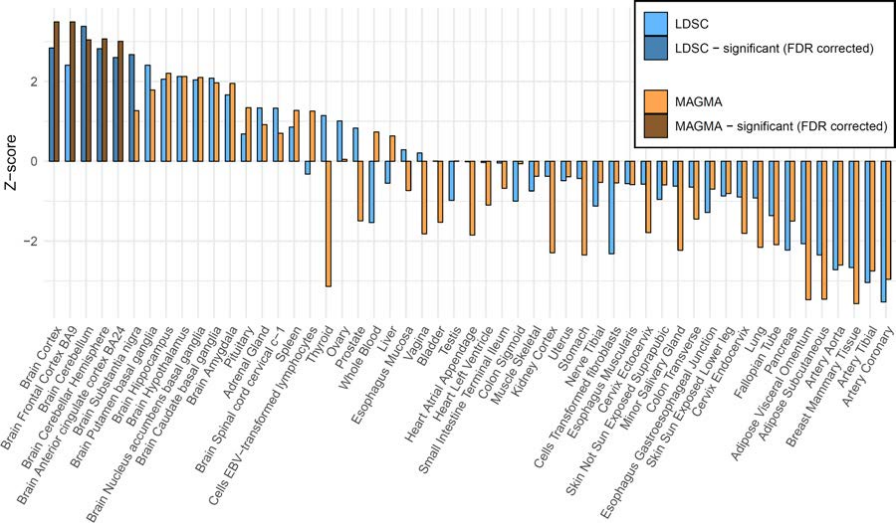
Enrichment of gene expression for 53 specific tissue types using MAGMA and LD-score regression.

First, we tested whether genome-wide effects on loneliness were consistent with tissue-specific differential gene expression based on GTEx RNA-sequence data from 53 tissues types using two approaches. For the first approach, we determined whether the distribution of effect sizes of all 17 715 protein-coding genes estimated from the gene-based tests showed enrichment of expression across multiple tissues (27). These results (Fig. 2) indicated that the gene-based association results were significantly enriched after FDR correction for genes with higher gene-expression levels in five brain tissues: cortex, cerebellum, cerebellar hemisphere, anterior cingulate cortex, and substantia nigra (with coefficients for the per-SNP contribution to the heritability ranging from 2.8 × 10^−9^ to 3.7 × 10^−9^). For the second approach, SNP heritability of loneliness was partitioned into categories of functional SNP annotations using LDSC (24). We found that SNPs associated with loneliness were also significantly more likely than expected by chance (after FDR correction) to regulate gene expression in five brain tissues: cortex, frontal cortex, cerebellum, cerebellar hemisphere, and anterior cingulate cortex (Fig. 2).

**Figure 3:**
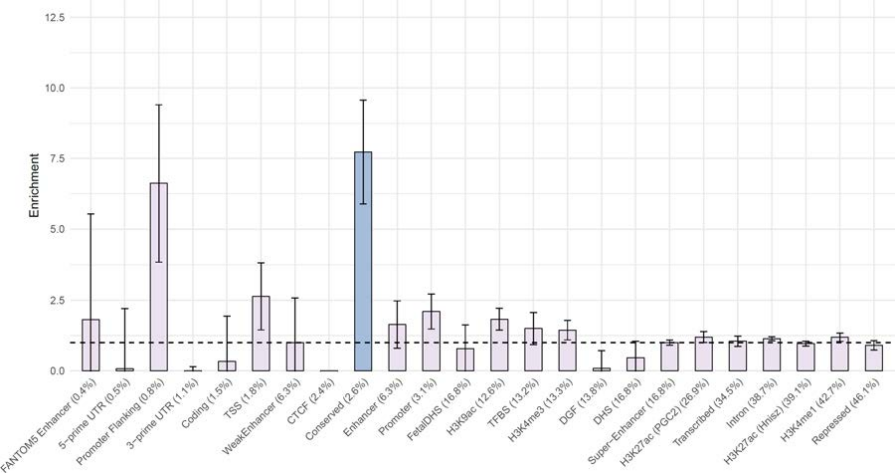
Enrichment of 24 annotations not specific to cell types, ordered by size (proportion of SNPs).

Second, we used LDSC to test for the enrichment of 24 genomic annotations that are not specific to any cell type, including coding vs non-coding regions, promoter regions, introns, and evolutionarily conserved regions (see Finucane et al, 2015 (28) for additional details). Of these 24 annotations, the genetic signals, after FDR correction, were significantly enriched for regions that were highly evolutionary conserved in mammals (similar to other polygenic traits) (28), which contain 2.6% of all SNPs but explain 20% of the loneliness heritability captured by all SNPs (Fig. 3).

### Genetic correlations

**Figure 4:**
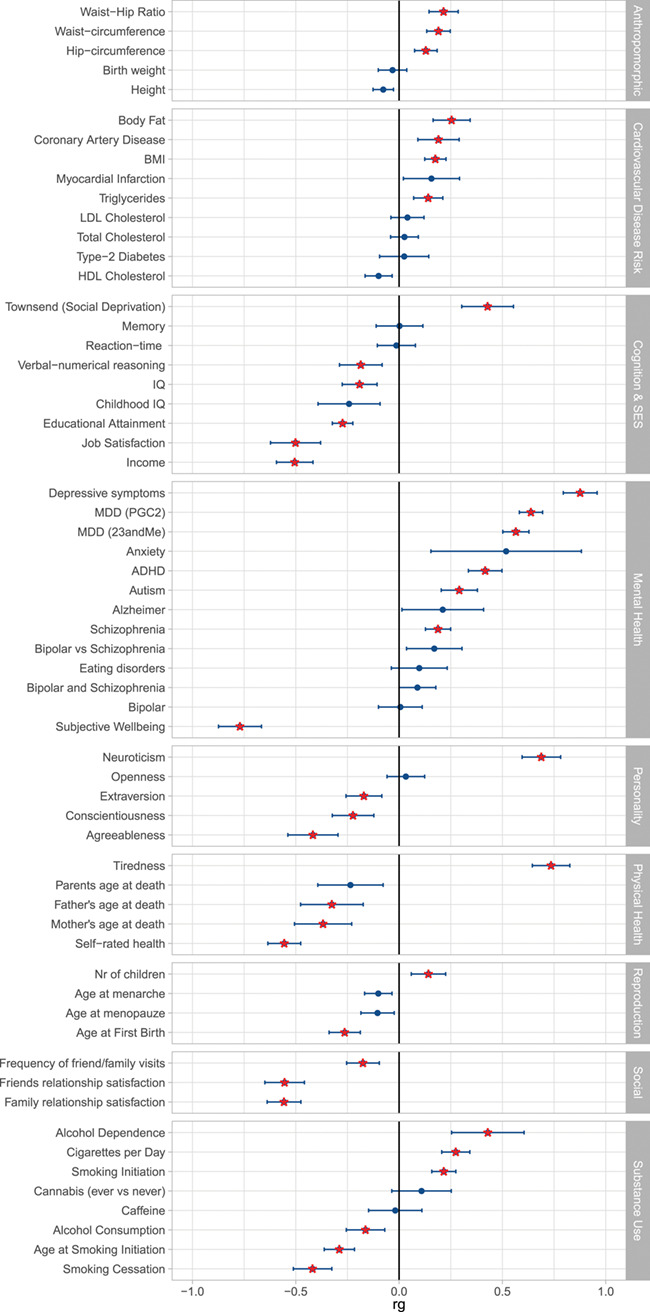
Genetic correlations as computed with LD-score regression. Red stars are significant after Bonferroni correction.

Genetic correlations (29) were estimated for loneliness and 61 characteristics from 9 domains including anthropomorphic traits, cardiovascular disease risk, cognitive functions, mental health, reproduction, and substance use. After applying a Bonferroni corrected significance threshold of 8.2 × 10^−4^, 39 out of 61 traits showed a significant genetic correlation with loneliness (Fig. 4 & Supplementary File 1). A significant signal was observed at least once from each of the 9 domains, with the strongest genetic correlations observed for mental health, especially for depressive symptoms (*r*_g_ = 0.88, *P* = 2.2 × 10^−101^), subjective wellbeing (*r*_g_ = −.77, *P* = 1 × 10^−49^), and MDD (*r*_g_ = 0.64, *P* = 5.2 × 10^−114^). In the health domain, tiredness and self-rated health showed the strongest correlations (*r*_g_ = 0.74, *P* = 3.2 × 10^−59^, and *r*_g_ = −.56, *P* = 2.5 × 10^−44^, respectively; more loneliness was correlated with more tiredness and worse health), while father’s and mother’s age of death showed modest but significant negative genetic correlations with loneliness (*r*_g_ = −.32, *P* = 1.8 × 10^−5^, and *r*_g_ = −.37, *P* = 1.1 × 10^−7^, respectively). Four out of five personality dimensions showed a significant genetic correlation with loneliness, with neuroticism showing the highest association (*r*_g_ = 0.69, *P* = 2.8 × 10^−49^); this genetic association was recently shown to be a major driver for the association between loneliness and personality (16). SES indicators related to economic success (Townsend index and income; *r*_g_ = 0.43, *P* = 7.7 × 10^−12^, and *r*_g_ = −.50, *P* = 3.4 × 10^−30^, respectively) and job satisfaction (*r*_g_ = − .0.50, *P* = 1.2 × 10^−16^) showed a considerably higher genetic correlation with loneliness than indicators of cognition (IQ and educational attainment; *r*_g_ = −.19, *P* = 5.9 × 10^−6^, and *r*_g_ = −0.27, *P* = 3.3 × 10^−28^, respectively). Genetic correlations with traits from the reproduction domain indicate that having more offspring and having offspring at a younger age is genetically associated with higher levels of loneliness, an association that is opposite to a previously reported phenotypic correlation (14). For substance use, alcohol consumption had a significant genetic correlation with loneliness (*r*_g_ = −0.16, *P* = 4.9 × 10^−4^), with more alcohol consumption is associated with lower loneliness, while alcohol dependence had a larger genetic correlation in the opposite direction (*r*_g_ = 0.43, *P* = 9.7 × 10^−7^). In the social circle domain, family and friendship satisfaction both showed significantly larger genetic correlations with loneliness (*r*_g_ = 0.56, *P* = 3.4 × 10^−42^, and *r*_g_ = 0.55, *P* = 5.7 × 10^−31^, respectively; more loneliness was associated with less family and friendship satisfaction) than the frequency of friend and family visits (*r*_g_ = 0.17, *P* = 1 × 10^−5^; more loneliness was associated with fewer visits), suggesting that the subjective experience of social isolation may play a larger role in feeling lonely than objective social isolation.

### PheWAS on the loneliness polygenic score

**Figure 5:**
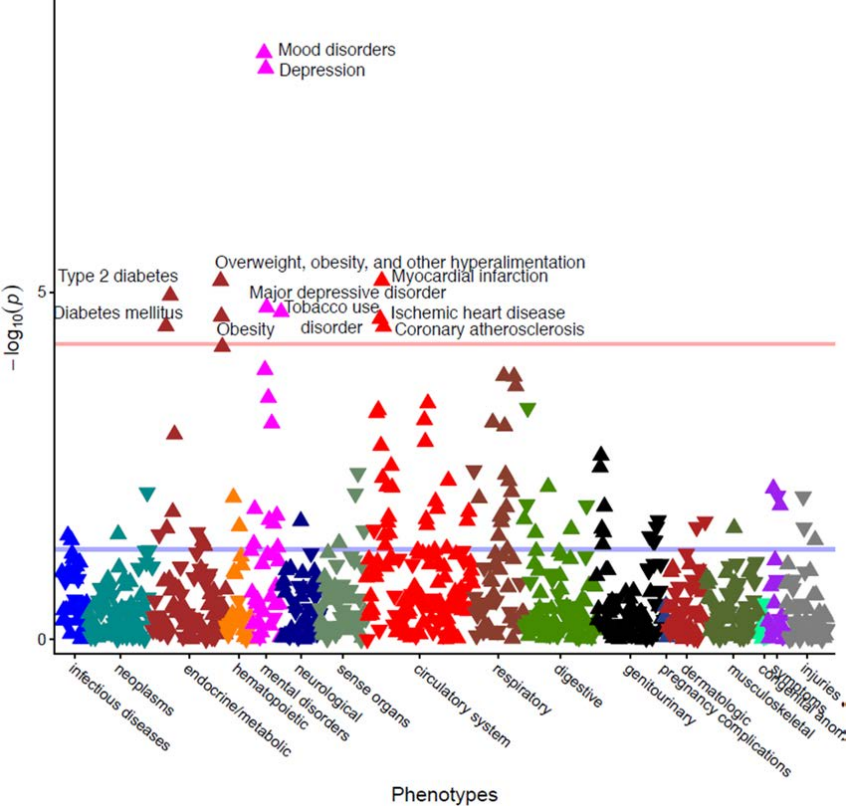
Results of the Phewas on the polygenic score for loneliness, corrected for gender, age, first 10 PCs, and batch.

Three cardiovascular, four neuropsychiatric, and four of the metabolic phenotypes were significantly associated with a genetic propensity to loneliness after Bonferroni correction for the 897 phenotypes tested (*P* < 5.57 × 10^−5^) (Fig. 5). Mood disorders yielded the most significant association with the loneliness polygenic score (*N*_cases_ = 3370, OR = 1.13, SE = 0.02, *P* = 3.57 × 10^−9^), followed by depression (*N*_cases_ = 3025, OR = 1.13, SE = 0.02, *P* = 5.98 × 10^−9^), myocardial infarction (*N*_cases_ = 2051, OR = 1.13, SE = 0.03, *P* = 6.82 × 10^−6^), overweight, obesity and other hyperalimentation (*N*_cases_ = 3040, OR = 1.10, SE = 0.02, *P* = 6.86 × 10^−6^), and type-2 diabetes (*N*_cases_ = 3967, OR = 1.09, SE = 0.02, *P* = 1.12 × 10^−5^). Complete results may be viewed interactively at https://sealockj.shinyapps.io/loneliness_interactive/.

In our subsequent analysis of quantitative lipid traits (with SNP-based heritabilities between ~5% and ~15%, see Supplementary Fig. 2), the loneliness polygenic score was modestly but significantly associated with reduced HDL (*R*^2^ = 0.12%, *P* = 5.69 x 10^−7^) and increased triglycerides (*R*^2^ = 0.27%, *P* = 2.43 x 10^−12^), but not pre-medication low-density lipoprotein (LDL) levels (*R*^2^ = 9.00 x 10^−4^%, *P* = 0.76; see Supplementary Table 7). To benchmark these results, we compared them to the proportion of variance explained by a polygenic score of corresponding lipids values (Supplementary Table 6) and for coronary artery disease (CAD) developed using the beta weights from the Global Lipids Genetics Consortium study (http://lipidgenetics.org/#DataDownloads) and the CARDIOoGRAMplusC4D study (http://www.cardiogramplusc4d.org/data-downloads/) (30). The proportion of variance explained by the polygenic score for CAD was similar in magnitude to the variance explained by the loneliness polygenic score for clinically evaluated HDL (*R*^2^ = 0.34%, *P* = 6.96 x 10^−10^), triglycerides (*R*^2^ = 0.22%, *P* = 7.52 x 10^−7^), and LDL-premed (*R*^2^ = 0.27%, *P* = 2.30 x 10^−5^; see Supplementary Fig. 3).

### Mendelian randomization

**Figure 6:**
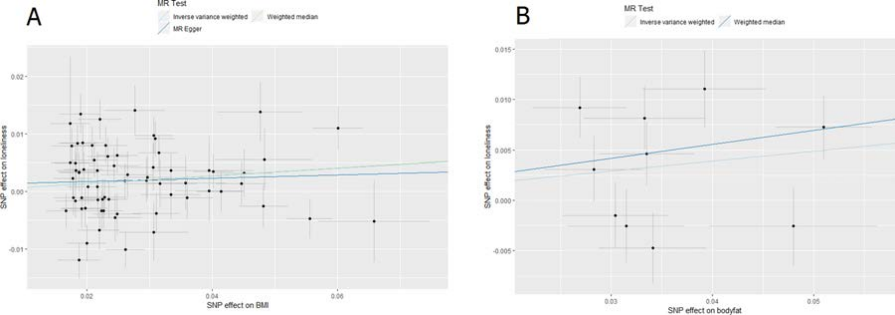
Two-Sample Mendelian Randomization results for the causal effect of (**A**) BMI on loneliness and (**B**) body fat on loneliness.

To test if the genetic correlations may reflect causality, we applied MR to examine evidence for causal effects of one phenotype on another. We made a selection of traits that showed a significant genetic correlation with loneliness and of which the top SNPs were unlikely to share pleiotropic effects with loneliness were examined. We focused on the relationship between loneliness and cardiovascular disease and its associated risk factors including CAD, myocardial infarction, HDL cholesterol, LDL cholesterol, total cholesterol, triglycerides, BMI, and body fat, which we also found to be significantly genetically correlated with loneliness. Of these traits, there were four with a significant genetic correlation with loneliness which we included in the MR analyses, namely CAD (*r*_g_ = 0.19), triglycerides (*r*_g_ = 0.14), BMI (*r*_g_ = 0.18), and body fat (*r*_g_ = 0.25) (see Fig. 6 and Supplementary Fig. 5). When both gene-exposure and gene-outcome associations are significant and in the expected ratio of a causal effect, and the MR assumptions are met (31), this is considered evidence for a causal relationship.

We found evidence of a causal, increasing the effect of BMI on loneliness using the inverse-variance weighted (IVW) median and GSMR methods, but not the MR-Egger method (Table 2 and Fig. 6A). The effect size of MR-Egger is of slightly weaker magnitude to the other two analyses but the Egger intercept is not significantly different from 0 (see Supplementary Table 8), indicating that there is no horizontal pleiotropy. There was evidence for heterogeneity between the different SNPs from Cochran’s Q (Supplementary Table 10). From body fat to loneliness, there was also evidence for a causal effect using the IVW, weighted median and GSMR methods (higher body fat caused more loneliness; Table 2 and Fig. 6B). Results of MR-Egger were not reported due to their limited reliability as shown by the low I^2^ statistic (0.57; Supplementary Table 9). Again, there was some evidence of heterogeneity between the different SNPs from Cochran’s Q. There was no clear evidence of causal effects of loneliness on any of the cardiovascular risk traits. We note that sample overlap between GWASs could cause a bias of MR results in the direction of the observational association. However, sample overlap was minimal in the present study (max 3.7%).

**Table 2:** Two sample, bidirectional Mendelian randomization results

Exposure	Outcome	*n*	IVW			Weighted median			MR-Egger			GSMR			
		SNPs	Beta	SE	*P*	Beta	SE	*P*	Beta	SE	*P*	SNPs	Beta	SE	*P*
Loneliness	BMI	9	−0.23	0.22	0.30	0.04	0.13	0.73	0.88^*^	1.72	0.62	8	0.02	0.13	0.87
Loneliness	Body fat	9	0.00	0.20	0.997	0.05	0.13	0.73	0.58^*^	1.71	0.74	8	0.25	0.17	0.14
Loneliness	Triglycerides	9	−0.06	0.17	0.72	0.02	0.14	0.90	n.a.	n.a.	n.a.	9	−0.09	0.14	0.53
Loneliness	CAD	12	−0.01	0.19	0.97	0.24	0.22	0.27	n.a.	n.a.	n.a.	13	0.14	0.23	0.54
BMI	Loneliness	53	0.03	0.03	0.22	0.02	0.03	0.59	0.04	0.07	0.61	65	**0.06**	**0.02**	**0.004**
Body fat	Loneliness	10	**0.10**	**0.04**	**0.01**	**0.14**	**0.05**	**0.003**	0.17^*^	0.25	0.52	10	**0.12**	**0.04**	**0.001**
Triglycerides	Loneliness	41	0.00	0.01	0.85	−0.01	0.02	0.75	−0.01	0.02	0.77	77	0.00	0.01	0.86
CAD	Loneliness	26	0.00	0.01	0.76	0.01	0.01	0.50	−0.02^*^	0.02	0.29	31	0.03	0.02	0.20

^*^Egger-SIMEX correction applied because of low *I*^2^ estimates. n.a. = *I*^2^ estimates too low to give reliable results for MR-Egger.

Bold = significance after Bonferroni corrections, with alpha = 0.05/8 = 0.00625

## Discussion

Chronic loneliness is strongly associated with physical and mental health and is a growing concern in many societies. In this study, we extended molecular genetic studies of loneliness by investigating the genetic architecture of loneliness and its relationship with a wide range of traits in 511 280 subjects of European ancestry from five Western countries, and an additional 18 498 US subjects of European ancestry in which we conducted a subsequent PheWAS. We identified 19 SNPs located in 16 independent loci that were significantly associated with loneliness. The most significant SNP signal came from chromosome 18 within the TCF4 gene, which plays an important role in nervous system development and has been associated with MDD (23). We detected 12 loci that were also detected by a previous study on social interaction and isolation that used the same UK Biobank subjects (17), and we report an additional four novel loci. In addition, we report 58 significantly associated gene from our genome-wide gene-based association analyses.

We found that the associated variants were significantly enriched for regions that are conserved in mammals, which has been observed for other polygenic traits as well (28). Additionally, we found that genome-wide signals were highly enriched for genes that were expressed in the brain, in particular in the cerebellum, (frontal) cortex, anterior cingulate cortex, and substantia nigra. The cerebellum is mostly known for its modulating role in motor, cognitive, and affective functions, and has been shown to play a role in social cognition as well, especially for processes that require higher-level abstraction away from the current event (i.e. past, future or hypothetical events) (32). The prefrontal cortex is implicated in the perception of social isolation (i.e. loneliness) (33–35). The anterior cingulate cortex is functionally connected with the prefrontal cortex, with which it is associated with emotional and physiological adjustments for potential threats and stressors, and is known to be involved the social (rather than the physical) pain associated with loneliness (36). The substantia nigra is best known for its role in reward and learning, which extends to social contexts as well (37). A large GWAS meta-analysis on MDD that included a similar tissue enrichment analyses identified only partly the same anatomical regions: all the same cortical regions were significantly associated with MDD (frontal cortex, cortex, anterior cingulate cortex, substantia nigra), but none of the cerebellar regions were (cerebellar hemisphere and cerebellum) (23). This suggests that the role of the cerebellar region may be more specific or larger for loneliness. Loneliness likely involves additional brain regions, since all 13 brain regions included in the enrichment analyses depicted in Figure 2 were nominally significant with *P* < 0.05.

Out of the 61 traits considered in our genetic correlation analyses, 39 showed a significant genetic correlation with loneliness, suggesting widespread shared genetic influences (e.g. pleiotropic effects) or causal relationships between loneliness and several traits. MDD has been strongly associated with loneliness in previous studies (38–40), but evidence from non-genetic longitudinal studies indicates that loneliness and depression are conceptually and statistically different constructs (39–41). Our results confirm the strong biological ties between loneliness, major depression, and depressive symptoms in both research ascertained samples and EHR from a hospital population. Our analyses do not provide conclusive findings however regarding the direction of causation in the relationship between loneliness and MDD, due to a lack of instrument variables for loneliness that are strong enough for causal inference.

There are several traits that show genetic correlations with the loneliness that are in opposite directions from previously reported phenotypic (non-genetic) correlations. Having more offspring was associated with lower levels of loneliness (14); however, the genetic correlations with number of offspring and age at first birth indicated that having more children or children at an earlier age was associated with more loneliness. Alcohol abuse has been associated with higher levels of loneliness (42); however we found a genetic correlation in the opposite direction with alcohol use. A possible explanation of these apparently contradictory results is that they are driven by a (genetic) association between loneliness and socio-economic status, which is related to many life outcomes. We observed a significant genetic overlap between loneliness and SES-related traits (e.g. income, job satisfaction, educational attainment, social deprivation of the neighborhood), with lower SES indicators showing a genetic association with more loneliness (with a particularly strong genetic correlation for income and job satisfaction: *r*_g_ = −.50). Number of offspring is negatively genetically correlated with educational attainment^43^ (and positively genetically correlated with loneliness), higher age at first birth is positively genetically correlated with educational attainment (43) (and negatively genetically correlated with loneliness), while alcohol consumption shows a positive genetic correlation with educational attainment (44) (and a negative genetic correlation with loneliness), and alcohol dependence is negatively genetically correlated with educational attainment (45) (and positively genetically correlated with loneliness); these observations are all in line with the negative genetic association between loneliness and educational attainment/SES (46).

Our phenome-wide analysis in a unique EHR dataset recapitulated the genetic correlation results and found that genetic propensity to loneliness is associated with increased risk for clinical depression, cardiovascular disease, and metabolic diseases such as type-2 diabetes. Elevated triglycerides and reduced HDL, two well-known risk factors for heart disease, were also associated with predisposition to loneliness after adjusting for covariates and even after restricting to levels prior to use of antilipemic medications. These findings provide a proof of principle that in clinical settings, polygenic scores may be used to uncover relationships between unmeasured behavioral traits (such as loneliness) and health outcomes. Another important advantage of this out-of-sample analysis is that by relying on clinical measurements and physician assigned ICD codes instead of retrospective self-report, we avoid potential reporting biases related to the loneliness that may influence correlations between loneliness and health outcomes. One possible limitation of this out-of-sample analysis could be the potential for some overfitting because we included all SNPs at a *P*-value threshold of 1. However, this is unlikely to be a substantial driver of our results given that an analysis of loneliness polygenic scores using a *P*-value threshold of 0.05 yielded similar findings (Supplementary Fig. 4). Another limitation is that while polygenic score analyses can identify novel genetic relationships, they cannot distinguish pleiotropy from causal effects.

With MR analysis, which can supply evidence for causal effects, we only found evidence of a causal, increasing, effect of BMI and body fat on loneliness. This concurs with a recent MR study reporting that BMI increases depressive symptoms and decreases subjective well-being (47). The causal effect size on loneliness was stronger for total body fat than for BMI, which may be due to body fat being a better measure for an unhealthy excess of body weight than BMI. Nonetheless, both findings point to an increased body weight causally leading to poorer mental health. Our MR analysis ruled out the possibility of horizontal pleiotropy among the instrumental variables in this analysis. It is important to note that the condition in MR of ‘no pleiotropy’ is only required for the instrument variables themselves and need not apply genome-wide. Indeed, it is possible (perhaps even likely) that the relationship between loneliness and health outcomes is influenced by bidirectional causal effects and pleiotropic biological effects.

In this study, we report 19 independent genetic associations in 16 loci and we report 58 genome-wide significant genes that are associated with loneliness in a sample of subjects from five Western countries. The genetic signals were enriched for genes expressed in specific brain tissues in cortical and cerebellar regions. We showed that the genetic risk for loneliness is associated with a wide range of health-related traits. Future work needs to establish the etiology of these associations and to determine which additional loci explain the rest of common genetic variation underlying loneliness, which together explained ~ 7% of individual differences.

## Materials and Methods

### Subjects & phenotype

A total of 511 280 adult subjects from 7 different cohorts were included in the GWAS meta-analysis. An overview of subjects and phenotyping across cohorts can be found in Supplementary Table 1. The UK Biobank (UKB) dataset was the largest. UKB was the only cohort with a dichotomous phenotype (N_total_ = 511 280: 81011 lonely and 367 945 non-lonely individuals). The other six cohorts had three types of continuous measures for loneliness: the sum of 9 items on a 4-point scale, the sum of 3 items on a 3-point scale, and 1 item on a 4-point scale.

### Genotyping and QC

Information on genotyping, imputation and QC is given in Supplementary Table 2. In all cohorts, SNP data were imputed to either 1000 Genomes or the Haplotype Reference Consortium (HRC). SNPs remaining after QC ranged from 5.7 million to 14.1 million. Based on ancestry information derived from SNP data, only subjects with European descent were included.

### GWASs & meta-analysis

GWASs were performed in all seven cohorts, with the variables age, sex, family relationships, and ancestry-informative PCs as fixed effects (see Supplementary Table 2 for details). The largest GWAS, the categorical GWAS for the UK Biobank dataset, was conducted using linear mixed modeling in fastGWA, which controls for both cryptic relatedness and population stratification (48, 49). The six continuous GWASs were meta-analyzed using the multivariate approach described in Baselmans et al (2019) (50). This approach controls for bias due to relatedness or sample overlap between GWASs by incorporating the cross-trait LD-score intercept (a measure for sample overlap) from LDSC (24) as weights. The categorical GWAS and continuous GWAS meta-analysis were then meta-analyzed using sample size-based weights in order to account for the respective differences of heritabilities, genetic correlation, and measurement scales of the categorical and continuous GWASs (see Demontis et al, 2017, for more details) (22).

### Follow-up analyses

Gene-based tests & gene enrichment tests: GWAS meta-analysis summary statistics were used to compute gene-based *P*-values in MAGMA (25) for 18 714 protein-coding genes using FUMA (27). MAGMA in FUMA was further used to test whether the effects of genes on loneliness were correlated with higher or lower gene expression in a given tissue based on GTEx RNA-seq data (27). This was tested for 53 specific tissue types.

LD-Score Regression Heritability Partitioning: Stratified LD-score regression was carried out using LDSC in order to partition the heritability signal into specific cell-type groups or genomic annotations (24,28). This method requires the GWAS meta-analysis summary statistics, and LD information based on an external reference panel, for which we used the European populations from the HapMap 3 reference panel.

S-PrediXcan: S-PrediXcan (26) uses reference panels with both measured gene expression and genotype data collected on the same individuals to build predictive models of gene expression in samples in which only genotype information is available. Predicted expression of genes for cases and controls can then be associated with phenotypic differences, yielding a test of association that incorporates transcriptional information. We used S-PrediXcan (26) to predict gene expression levels in 10 brain tissues and to test whether the predicted gene expression correlates with loneliness. Pre-computed tissue weights were employed from the Genotype-Tissue Expression (GTEx v7) project database (https://www.gtexportal.org/) (51) as the reference transcriptome dataset. As input data, we included the loneliness GWAS meta-analysis summary statistics, transcriptome tissue data, and covariance matrices of the SNPs within each gene model (based on HapMap SNP set; available to download at the PredictDB Data Repository) from 10 brain tissues: anterior cingulate cortex, caudate basal ganglia, cerebellar hemisphere, cerebellum, cortex, frontal cortex, hippocampus, hypothalamus, nucleus accumbens basal ganglia, and putamen basal ganglia. We used a transcriptome-wide significant threshold of *P* < 1.29 × 10^−6^, which is the Bonferroni corrected threshold when adjusting for all tissues and genes (38 611 gene-based tests).

Genetic correlations: Genetic correlations between loneliness and 61 other traits were computed in LDSC (29). Here, the genetic correlation between traits is based on the estimated slope from the regression of the product of z-scores from two GWASs on the LD score and represents the genetic covariation between the two traits based on all polygenic effects captured by the included SNPs. Summary statistics from well-powered GWASs were available for 61 traits related to personality, cognition, reproduction, social circle, body composition, substance use, and physical and mental health. Multiple testing was corrected for using a Bonferroni corrected significance threshold of.05/61 = 8.2 × 10^−4^. LD scores were based on European populations from the HapMap 3 reference panel (24,29).

Polygenic scores for the PheWAS: Since loneliness is not a phenotype systematically documented within the medical record, we were unable to determine the best fit *P*-value threshold for polygenic scoring. Therefore, we relied on an inclusive threshold (*P* < 1) and applied the clumping protocol to all SNPs to generate polygenic scores. All SNPs from the loneliness meta-analysis were thinned using an association-driven pruning algorithm that clumped SNPs into 250 kb windows and removed SNPs in LD (*r*^2^ > 0.1) with the most associated SNP (i.e. lowest *P*-value) in that window. LD estimates were directly derived from the BioVU samples (see below). After clumping, a total of 93 501 LD-independent SNPs remained for scoring. Scores were then constructed using PRSice software (52) and defined by the sum of the number of risk alleles at each locus, weighted by their estimated effect sizes. The polygenic scores were calculated in an independent sample of 18 498 genotyped individuals of European descent in BioVU. Genotyping and QC of this sample have been described elsewhere (20,53).

PheWAS: In the genotyped BioVU sample, a logistic regression model was fitted to each of 897 case/control phenotypes to estimate the odds of each diagnosis given the loneliness polygenic score, after adjustment for sex, median age of the longitudinal HER measurements, top 10 principal components of ancestry, and genotyping batch. The 897 disease phenotypes included 32 infectious diseases, 75 neoplasms, 86 endocrine/metabolic diseases, 29 hematopoietic diseases, 36 mental disorders, 44 neurological disorders, 54 sense organs, 126 circulatory system disorders, 59 respiratory diseases, 85 digestive diseases, 77 genitourinary diseases, 3 pregnancy complications, 43 dermatologic disorders, 64 musculoskeletal disorders, 8 congenital anomalies, 24 symptoms, and 52 injuries/poisonings. We required the presence of at least two International Classification of Disease (ICD) codes that mapped to a PheWAS disease category (Phecode Map 1.2 (https://phewascatalog.org/phecodes) to assign ‘case’ status. PheWAS analyses were run using the PheWAS R package (54).

Lipid traits in the EHR: We examined the relationship between the loneliness polygenic score and three quantitative lipid traits. Clinically measured lipid levels included LDL (*N* = 6455 with pre-medication values), high-density lipoprotein (HDL) (*N* = 10 722), and triglycerides (trigs) (*N* = 11 012; Supplementary Table 5). As most patients had multiple lipid values available in their EHRs, we calculated median LDL, HDL, and triglyceride values for each patient after removing outlier values that were +/− 4 SDs from the sample mean. To adjust for age, we extracted the age at the median lipid value. If the number of lab measurements was even, we used the average age between the two median measurements. We then regressed the median lab value on sex and the cubic spline of median age, and quantile normalized the residuals. For sensitivity analyses, we also calculated the median of pre-medication (Supplementary Table 5) lipid values, using only observations that occurred before the first mention of lipid-lowering medication in the EHR (55), and transformed the age- and sex-adjusted residuals as above. Linear regression models were then fitted to the median LDL, HDL, and trigs values respectively to estimate the effect of the loneliness polygenic score on each lipid trait. As the lipid traits were already sex and age-adjusted, we included only the top 10 principal components of ancestry and genotyping batch as covariates.

*MR*: We performed two-sample bidirectional MR (56) analyses to investigate the direction of causality in the relationship between loneliness and cardiovascular risk factors and diseases. Of the eight cardiovascular risk factors and diseases for which we know the genetic correlations from the LDSC analyses (CAD, myocardial Infarction, HDL cholesterol, LDL cholesterol, total cholesterol, triglycerides, BMI and body fat), we tested the four traits that showed a significant genetic correlation, namely CAD (*r*_g_ = 0.19), triglycerides (*r*_g_ = 0.14), BMI (*r*_g_ = 0.18), and body fat (*r*_g_ = 0.25). We used genome-wide significant SNPs from the five GWASs (loneliness and the four significant traits) to serve as instrumental variables (gene-exposure association). SNPs were pruned for LD (*r*^2^ < 0.001), and the remaining SNPs (or proxy SNPs with *r*^2^ ≥ 0.8 when the top-SNP was not available in the other GWASs) were then identified in the GWAS summary statistics of the outcome variable (gene-outcome association). When both gene-exposure and gene-outcome associations are significant and in the expected ratio of an indirect causal effect, and the MR assumptions are met (31), this is considered the evidence for a causal relationship. We combined estimates from individual SNPs by applying IVW linear regression (57). We conducted three sensitivity analyses more robust to horizontal pleiotropy (i.e. effects of the SNPs on the target outcome outside of their effect on the exposure), each relying on distinct assumptions: weighted median regression (58), MR-Egger regression (59) and Generalized Summary-data based Mendelian Randomization (GSMR) (60). Weighted median regression can provide a consistent estimate of a possible causal effect, even when up to 50% of the weight in the genetic instrument comes from invalid instruments. MR-Egger regression uses ‘Egger’s test’ to test for bias from horizontal pleiotropy. MR-Egger will provide a consistent estimate of the causal effect, given that the strength of the genetic instrument (gene-exposure association) does not correlate with the effect that the instrument has on the outcome. This InSIDE assumption (Instrument Strength Independent of Direct Effect) is a much weaker assumption than the assumption that there is no pleiotropy. However, if the no measurement error (NOME) assumption is violated, MR-Egger may be biased. Violation of NOME can be assessed with the I^2^ statistic, which ranges between 0 and 1. When I^2^ is below 0.9, there is a considerable risk of bias. By applying MR-Egger simulation extrapolation (SIMEX) (61), this bias can be corrected for. When I^2^ is below 0.6 the results of MR-Egger (even with SIMEX correction) are not reliable. For our analyses we report MR-Egger results when I^2^ > 0.9, MR-Egger-SIMEX results when I^2^ = 0.6–0.9 and we don’t report MR-Egger results when I^2^ < 0.6. Lastly, we performed GSMR, a method that takes into account LD between the different genetic variants included in an instrument. Since GSMR accounts for LD, we pruned the genetic variants included in GSMR instruments at a higher threshold of *r*^2^ < 0.05 (as opposed to *r*^2^ < 0.001). Including SNPs in higher LD than 0.05 was shown to provide very limited increase in power. GSMR includes a filtering step which excludes SNPs that are suspected to have pleiotropic effects on both the exposure and the outcome (HEIDI filtering).

## Funding

The National Institutes of Health (NIH, R01AG033590 to JC); the Royal Netherlands Academy of Science Professor Award (PAH/6635 to NTR: DIB). Data collection and genotyping in NTR by the Netherlands Organization for Scientific Research (904-61-090, 85-10-002, 904-61-193, 480-04-004, 400-05-717, Spi-56-464-14192 and 480-15-001/674); Biobanking and Biomolecular Resources Research Infrastructure (BBMRI – NL, 184.021.007 and 184.033.111); the Avera Institute for Human Genetics, Sioux Falls, South Dakota (USA) and the National Institutes of Health (NIH, R01D0042157-01A); the NIMH Grand Opportunity grants (1RC2MH089951-01 and 1RC2 MH089995-01). A Rubicon grant from the Netherlands Organization for Scientific Research (NWO; grant number 446-16-009 to J.L.T.); NIMH (grant 5R01MH113362-02 to L.K.D.); NIH training grant (2T32GM080178 to J.M.S). The Frontiers of Innovation Scholars Program (FISP; #3-P3029 to S.S.-R.); the Interdisciplinary Research Fellowship in NeuroAIDS (IRFN; MH081482); a pilot award from DA037844 and 2018 NARSAD Young Investigator Grant (27676); the California Tobacco-Related Disease Research Program (TRDRP; Grant Number 28IR-0070 to S.S.-R. and A.A.P.); Institutional funding (the 1S10RR025141-01 instrumentation award, and by the CTSA grant UL1TR000445 from NCATS/NIH to Medical Center’s BioVU); NIH (additional funding through grants P50GM115305 and U19HL065962); Part of the computations for this paper was performed on Cartesius (grant ‘Population scale genetic analysis’; NWO rekentijd: 16332).

## Conflict of Interest Statement

PF, SLE and members of the 23andMe research team are employees of 23andMe Inc.

## Data access

The full summary statistics for the 23andMe dataset will be made available to qualified investigators who enter into an agreement with 23andMe that protects participant privacy. Interested investigators should visit research.23andMe/collaborate/#publication to learn more and to apply for access.

## Supplementary Material

SUPPLEMENTARY_FILE_HUM_MOL_GEN_revision_02092019_ddz219

SUPPLEMENTARY_loneliness_GWAS_HUM_MOL_GEN_revision_02092019_ref_to_text_ddz219
