# Supplementary material for: Phenome-wide investigation of health outcomes associated with genetic predisposition to loneliness
Source: Hum Mol Genet. 2019 Sep 13;28(22):3853–65. doi: 10.1093/hmg/ddz219 (PMC6935385; doi:10.1093/hmg/ddz219)

Supplementary Table 1: Subjects and phenotype details per cohort

| **Dataset** | **Sample Size** | **% Females** | **Mean Age (SE)** | **Loneliness Measure** |
| --- | --- | --- | --- | --- |
| UK Biobank^63^ | 511,280 | 54% | 56.5 (8.1) | Do you often feel lonely? (yes/no) |
| 23andMe^64^ | 20,591 | 55% | 59.6 (16.0) | 9-item questionnaire (4-point scale):   1. I feel in tune with the people around me 2. There are people I can turn to. 3. I feel alone 4. I feel part of a group of friends 5. I have a lot in common with the people around me 6. I feel isolated from others 7. There are people who really understand me 8. I am unhappy being so withdrawn 9. There are people I can talk to |
| Netherlands Twin Register^65^ | 11,046 | 63% | 44.6 (17.1) | 3-item questionnaire (3-point scale):   1. How often do you feel left out? 2. How often do you feel isolated from others? 3. How often do you feel that you lack companionship? |
| Health & Retirement Study^15^ | 7,556 | 59% | 67.2 (10.3) | 3-item questionnaire (3-point scale):   1. How often do you feel left out? 2. How often do you feel isolated from others? 3. How often do you feel that you lack companionship? |
| Rotterdam Study^66^ | 7,764 | 57% | 67 (10.7) | Did you feel lonely during the past week? (4-point scale) |
| Sweden – SALTY^67^ | 5,750 | 52.2% | 49.8 (4.2) | Did you feel lonely during the past week? (4-point scale) |
| Sweden – TwinGene^67^ | 9,617 | 53% | 58.4 (8.0) | Did you feel lonely during the past week? (4-point scale) |

Supplementary Table 2: Genotyping, imputation, QC, and GWAS information per cohort

| **Dataset** | **Microarray(s)** | **Imputation Panel** | **SNP MAF, INFO, HWE exclusion thresholds** | **N SNPs after QC** | **Identification of European Ancestry** | **GWAS analysis** |
| --- | --- | --- | --- | --- | --- | --- |
| UK Biobank^63^ | UK BiLEVE array & UK Biobank Axiom Array | Haplotype Reference Consortium | MAF <.0001;  INFO <.8; HWE *p* < 10^-6^ | 12,602,501 | Self-report & PCA | Linear mixed modeling in fastGWA.^48^ Covariates: sex, age, 20 PCs. |
| 23andMe^64^ | 23andMe custom genotyping array platforms^62^ | 1000 Genomes (Phase 1 Version 3) | MAF <.01;  INFO <.5; HWE *p* < 10^-20^ | 14,113,458 | Ancestry Composition^68^ | Linear regression using the internal 23andMe pipeline. ^64^  Covariates: age, sex, 5 PCs, genotype platform. |
| Netherlands Twin Register^65^ | Several Illumina and Affymetrix platforms^63,67^ | Haplotype Reference Consortium | MAF <.01;  INFO <.4; HWE *p* < 10^-5^ | 6,917,809 | PCA | PLINK linear regression with within family clustering. Covariates: sex, age, 10 PCs |
| Health & Retirement Study^15^ | Illumina Human Omni-2.5 | Haplotype Reference Consortium | MAF <.01;  INFO <.5; HWE *p* < 10^-6^ | 5,768,559 | NA | Linear mixed model in GEMMA.  Covariates: sex, age, marital status |
| Rotterdam Study^66^ | Several Illumina and Affymetrix platforms^64^ | Haplotype Reference Consortium | MAF <.05;  INFO <.4;  HWE *p* <10^-7^ | 6,984,254 | PCA | Linear mixed model in GCTA. Covariates: sex, age, 10 PCs |
| Sweden – SALTY^67^ | Illumina Infinium PsychArray-24 | 1000 Genomes (Phase 1 Version 3) | MAF < .01;  INFO < .8; HWE *p* < 10^-6^ | 8,511,408 | PCA | Linear mixed model in RAREMETALWORKER. Covariates: sex, age, 10 PCs |
| Sweden – TwinGene^67^ | Illumina OmniExpress | 1000 Genomes (Phase 1 Version 3) | MAF < .01;  INFO < .8; HWE *p* < 10^-6^ | 8,834,367 | PCA | PLINK linear regression with within family clustering. Covariates: sex, age, 10 PCs |

*Supplementary Table 3: Sample size, lambda, intercept, and h2 estimated from LD score regression*

|  | **UKB** | **23andMe** | **HRS** | **NTR** | **Rotterdam** | **Sweden - SALTY** | **Sweden - TwinGene** |
| --- | --- | --- | --- | --- | --- | --- | --- |
| *N* | 511,280 | 20,591 | 7,556 | 11,046 | 7,764 | 5,750 | 9,617 |
| *Lambda* | 1.30 | 1.047 | 1.017 | 1.002 | 1.002 | 1.011 | 1.011 |
| *Intercept* | 1.017 (.008) | 1.001 (.006) | 1.006 (.006) | 1.005 (.006) | .994 (.006) | .99 (.007) | 1.020 (.006) |
| *h^2^* | .081 (.01) | .096 (.02) | .106 (.06) | .002 (.04) | .031 (.06) | .108 (.08) | -.047 (.04) |

Supplementary Table 4: Significant associations of S-PrediXcan gene-based association analyses (Bonferroni corrected significance threshold = 0.05/38611 = 1.29 × 10^-6^)

| **Gene** | **Gene name** | **Z score** | **Effect Size** | ***p*-value** | **Var g** | **Pred perf r2** | **Pred perf pval** | **N SNPs used** | **N SNPs in cov** | **N snps in model** | **Tissue** |
| --- | --- | --- | --- | --- | --- | --- | --- | --- | --- | --- | --- |
| ENSG00000249484 | *AC091969.1* | 6.105 | 0.063 | 1.03E-09 | 0.044 | 0.112 | 1.25E-04 | 31 | 31 | 31 | Caudate |
| ENSG00000109919 | *MTCH2* | -5.766 | -0.069 | 8.10E-09 | 0.031 | 0.044 | 1.54E-02 | 19 | 19 | 19 | Cerebellum |
| ENSG00000225190 | *PLEKHM1* | 5.391 | 0.101 | 7.01E-08 | 0.014 | 0.153 | 7.31E-05 | 10 | 12 | 12 | Anterior cingulate cortex |
| ENSG00000262539 | *RP11-259G18.3* | 5.245 | 0.077 | 1.56E-07 | 0.023 | 0.333 | 6.89E-17 | 29 | 56 | 56 | Caudate |
| ENSG00000109919 | *MTCH2* | -5.241 | -0.044 | 1.60E-07 | 0.053 | 0.118 | 2.91E-05 | 18 | 18 | 18 | Caudate |
| ENSG00000233276 | *GPX1* | 5.197 | 0.026 | 2.03E-07 | 0.182 | 0.215 | 1.45E-07 | 54 | 55 | 55 | Prefrontal cortex |
| ENSG00000263503 | *RP11-707O23.5* | 5.191 | 0.035 | 2.09E-07 | 0.121 | 0.504 | 1.92E-17 | 15 | 22 | 22 | Hippocampus |
| ENSG00000262500 | *RP11-259G18.2* | 5.191 | 0.072 | 2.09E-07 | 0.035 | 0.527 | 3.17E-17 | 11 | 23 | 23 | Putamen |
| ENSG00000262500 | *RP11-259G18.2* | 5.184 | 0.061 | 2.17E-07 | 0.034 | 0.475 | 6.68E-15 | 12 | 20 | 20 | Hypothalamus |
| ENSG00000165915 | *SLC39A13* | -5.180 | -0.028 | 2.22E-07 | 0.145 | 0.168 | 1.77E-05 | 20 | 20 | 20 | Prefrontal cortex |
| ENSG00000185829 | *ARL17A* | 5.168 | 0.033 | 2.37E-07 | 0.132 | 0.295 | 2.77E-10 | 21 | 29 | 29 | Nucleus accumbens |
| ENSG00000261575 | *RP11-259G18.1* | 5.139 | 0.050 | 2.76E-07 | 0.067 | 0.365 | 2.04E-13 | 21 | 47 | 47 | Cortex |
| ENSG00000184922 | *FMNL1* | -5.129 | -0.026 | 2.91E-07 | 0.222 | 0.292 | 7.06E-13 | 372 | 374 | 374 | Cerebellum |
| ENSG00000214425 | *LRRC37A4P* | -5.116 | -0.016 | 3.12E-07 | 0.518 | 0.623 | 1.07E-27 | 330 | 332 | 332 | Hemisphere |
| ENSG00000261575 | *RP11-259G18.1* | 5.108 | 0.022 | 3.26E-07 | 0.316 | 0.556 | 1.29E-27 | 36 | 44 | 44 | Cerebellum |
| ENSG00000264070 | *DND1P1* | 5.090 | 0.050 | 3.57E-07 | 0.044 | 0.377 | 1.65E-12 | 16 | 24 | 24 | Prefrontal cortex |
| ENSG00000262539 | *RP11-259G18.3* | 5.089 | 0.024 | 3.60E-07 | 0.239 | 0.513 | 1.40E-19 | 18 | 24 | 24 | Nucleus accumbens |
| ENSG00000262539 | *RP11-259G18.3* | 5.084 | 0.032 | 3.69E-07 | 0.140 | 0.579 | 7.78E-30 | 31 | 44 | 44 | Cerebellum |
| ENSG00000238083 | *LRRC37A2* | 5.058 | 0.022 | 4.23E-07 | 0.297 | 0.638 | 1.46E-34 | 33 | 40 | 40 | Cerebellum |
| ENSG00000173531 | *MST1* | -5.057 | -0.025 | 4.25E-07 | 0.175 | 0.050 | 1.11E-02 | 55 | 65 | 65 | Cerebellum |
| ENSG00000196666 | *FAM180B* | -5.046 | -0.035 | 4.50E-07 | 0.078 | 0.076 | 1.53E-03 | 26 | 26 | 26 | Cerebellum |
| ENSG00000225190 | *PLEKHM1* | -5.043 | -0.019 | 4.59E-07 | 0.462 | 0.562 | 1.02E-28 | 43 | 45 | 45 | Cerebellum |
| ENSG00000263503 | *RP11-707O23.5* | 5.013 | 0.088 | 5.37E-07 | 0.015 | 0.438 | 1.11E-15 | 7 | 18 | 18 | Prefrontal cortex |
| ENSG00000263503 | *RP11-707O23.5* | 4.993 | 0.036 | 5.94E-07 | 0.102 | 0.591 | 4.17E-26 | 17 | 36 | 36 | Cortex |
| ENSG00000233276 | *GPX1* | 4.987 | 0.020 | 6.14E-07 | 0.258 | 0.245 | 3.36E-09 | 67 | 68 | 68 | Caudate |
| ENSG00000262500 | *RP11-259G18.2* | 4.968 | 0.025 | 6.76E-07 | 0.216 | 0.547 | 1.87E-19 | 34 | 39 | 39 | Anterior cingulate cortex |
| ENSG00000263503 | *RP11-707O23.5* | 4.962 | 0.030 | 6.98E-07 | 0.135 | 0.554 | 7.83E-27 | 9 | 16 | 16 | Cerebellum |
| ENSG00000233276 | *GPX1* | 4.961 | 0.035 | 7.03E-07 | 0.090 | 0.183 | 1.46E-05 | 74 | 74 | 74 | Anterior cingulate cortex |
| ENSG00000185829 | *ARL17A* | 4.956 | 0.025 | 7.19E-07 | 0.204 | 0.600 | 4.74E-31 | 24 | 37 | 37 | Cerebellum |
| ENSG00000204650 | *CRHR1-IT1* | 4.944 | 0.067 | 7.66E-07 | 0.030 | 0.486 | 1.10E-17 | 9 | 24 | 24 | Putamen |
| ENSG00000186868 | *MAPT* | -4.942 | -0.058 | 7.73E-07 | 0.043 | 0.076 | 1.56E-03 | 11 | 16 | 16 | Cerebellum |
| ENSG00000172247 | *C1QTNF4* | 4.941 | 0.033 | 7.79E-07 | 0.102 | 0.111 | 5.64E-04 | 46 | 46 | 46 | Hemisphere |
| ENSG00000196666 | *FAM180B* | -4.923 | -0.034 | 8.54E-07 | 0.080 | 0.140 | 1.24E-04 | 11 | 12 | 12 | Prefrontal cortex |
| ENSG00000263503 | *RP11-707O23.5* | 4.895 | 0.021 | 9.81E-07 | 0.230 | 0.496 | 4.93E-17 | 39 | 50 | 50 | Anterior cingulate cortex |
| ENSG00000225190 | *PLEKHM1* | -4.893 | -0.020 | 9.94E-07 | 0.340 | 0.567 | 6.87E-23 | 31 | 38 | 38 | Hemisphere |
| ENSG00000204650 | *CRHR1-IT1* | 4.891 | 0.040 | 1.00E-06 | 0.082 | 0.385 | 5.17E-13 | 23 | 35 | 35 | Anterior cingulate cortex |
| ENSG00000184922 | *FMNL1* | -4.887 | -0.025 | 1.02E-06 | 0.197 | 0.389 | 7.77E-15 | 26 | 28 | 28 | Hemisphere |
| ENSG00000109919 | *MTCH2* | -4.873 | -0.041 | 1.10E-06 | 0.056 | 0.140 | 8.06E-05 | 30 | 30 | 30 | Prefrontal cortex |
| ENSG00000225190 | *PLEKHM1* | 4.872 | 0.049 | 1.11E-06 | 0.053 | 0.120 | 2.27E-04 | 12 | 13 | 13 | Nucleus accumbens |
| ENSG00000204650 | *CRHR1-IT1* | 4.869 | 0.026 | 1.12E-06 | 0.228 | 0.398 | 1.56E-14 | 21 | 25 | 25 | Cortex |
| ENSG00000185829 | *ARL17A* | 4.867 | 0.024 | 1.13E-06 | 0.214 | 0.433 | 6.96E-14 | 38 | 50 | 50 | Prefrontal cortex |
| ENSG00000214425 | *LRRC37A4P* | -4.860 | -0.030 | 1.17E-06 | 0.138 | 0.356 | 3.49E-15 | 413 | 428 | 428 | Hypothalamus |
| ENSG00000263503 | *RP11-707O23.5* | 4.859 | 0.034 | 1.18E-06 | 0.095 | 0.565 | 3.93E-23 | 18 | 33 | 33 | Hemisphere |
| ENSG00000214425 | *LRRC37A4P* | -4.854 | -0.020 | 1.21E-06 | 0.352 | 0.530 | 3.58E-28 | 433 | 438 | 438 | Cerebellum |
| ENSG00000263503 | *RP11-707O23.5* | 4.852 | 0.046 | 1.22E-06 | 0.070 | 0.478 | 1.30E-19 | 13 | 22 | 22 | Nucleus accumbens |
| ENSG00000214425 | *LRRC37A4P* | -4.845 | -0.020 | 1.27E-06 | 0.339 | 0.574 | 2.09E-23 | 20 | 23 | 23 | Nucleus accumbens |
| ENSG00000185829 | *ARL17A* | 4.841 | 0.025 | 1.29E-06 | 0.191 | 0.409 | 2.15E-16 | 20 | 23 | 23 | Caudate |

*Gene name = as listed by the Transcriptome Model, generally extracted from Genquant (http://www.gencodegenes.org/); Z score = summary PrediXcan's association*

*Supplementary Table 5. Characteristics of genotyped BioVU patients with lipid measurements*

|  | HDL | LDL | TG |
| --- | --- | --- | --- |
| Number of individuals | 10722 | 10492 | 11012 |
| Number of observations | 74171 | 68737 | 77812 |
| Median lab value in mg/dL, mean (sd) | 49.1 (17.3) | 99.7 (32.6) | 149.7 (92.3) |
| Age in years at median lab value, mean (sd) | 59.4 (13.8) | 59.6 (13.8) | 59.1 (13.9) |
| Multiple observations |  |  |  |
| Number of individuals with >1 observation (%) | 8160 (76.1) | 7918 (75.5) | 8366 (76.0) |
| Number of observations per individual, mean (sd) | 8.8 (8.5) | 8.4 (8.1) | 9.0 (9.4) |
| Number of years between first and last observations, mean (sd) | 7.7 (5.4) | 7.3 (5.0) | 7.6 (5.4) |
| Lab value range within an individual, mean (sd) | 20.1 (14.4) | 57.3 (39.6) | 147.2 (145.0) |
| Median absolute deviation within an individual, mean (sd) | 6.5 (4.9) | 18.4 (13.5) | 44.5 (44.2) |
| Anti-lipemic medications |  |  |  |
| Number of individuals with pre-medication lab values (%) | 6742 (62.9) | 6455 (61.5) | 7060 (64.1) |
| Number of pre-medication observations ( % of all observations) | 23686 (31.9) | 21434 (31.2) | 26939 (34.6) |
| Median of pre-medication lab value in mg/dL, mean (sd) | 52.0 (18.3) | 115.2 (35.2) | 153.0 (101.5) |

*Supplementary Table 6. Validation of EHR-derived lipid values. Polygenic scores for HDL, LDL, and TG constructed from SNPs below a p-value threshold of 5x10^-8^ in the discovery sample were associated with the same trait in BioVU.*

| Phenotype | R^2^ | P | N SNPs |
| --- | --- | --- | --- |
| HDL | 0.056 | 1.29E-140 | 186 |
| HDL-premed | 0.057 | 3.18E-91 | 186 |
| LDL | 0.017 | 4.43E-42 | 80 |
| LDL-premed | 0.029 | 7.89E-43 | 80 |
| TG | 0.044 | 8.79E-110 | 124 |
| TG-premed | 0.046 | 3.60E-75 | 124 |

*Supplementary Table 7. Prediction of lipid levels in BioVU using polygenic scores for CAD and loneliness. We identified the best fit p-value threshold for each trait pair by iterating over thresholds from 5 × 10-8 to 0.5 in increments of 5 × 10-4. R^2^ is the proportion of variance in the trait explained by the polygenic score, p-value is its strength of association, and N SNPs denotes the number of SNPs included in the polygenic score at a given p-value threshold.*

| **Base** | **Phenotype** | **Threshold** | **R^2^** | ***p*-value** | **N SNPs** |
| --- | --- | --- | --- | --- | --- |
| CAD | HDL | 1 | 0.00343 | 6.96E-10 | 121732 |
| CAD | LDL-premed | 5.00E-08 | 0.00277 | 2.30E-05 | 42 |
| CAD | TG | 0.0169501 | 0.00220 | 7.52E-07 | 7636 |
| loneliness | HDL | 1 | 0.0012 | 5.69E-7 | 94,785 |
| loneliness | LDL-premed | 1 | 9.00E-6 | 0.76 | 94,785 |
| loneliness | TG | 1 | 0.00274 | 2.43E-12 | 94,785 |

*Supplementary Table 8: MR-Egger intercept, indicating horizontal pleiotropy, for bidirectional two-sample Mendelian randomization analyses*

| Exposure | Outcome | *n* | Egger intercept | | |
| --- | --- | --- | --- | --- | --- |
|  |  | SNPs | intercept | *SE* | *p* |
| Loneliness | BMI | 13 | *n.a.* | *n.a.* | *n.a.* |
| Loneliness | Body fat | 13 | *n.a.* | *n.a.* | *n.a.* |
| Loneliness | Triglycerides | 12 | *n.a.* | *n.a.* | *n.a.* |
| Loneliness | CAD | 15 | *n.a.* | *n.a.* | *n.a.* |
| BMI | Loneliness | 68 | 0.001 | 0.002 | 0.564 |
| Body fat | Loneliness | 10 | *n.a.* | *n.a.* | *n.a.* |
| Triglycerides | Loneliness | 54 | 0.000 | 0.000 | 0.516 |
| CAD | Loneliness | 38 | -0.001 | 0.002 | 0.592 |

n.a. = I^2^ estimates too low to give reliable results for MR-Egger.

*Supplementary Table 9: I-squared statistic*

| Exposure | Outcome | *n* |  |
| --- | --- | --- | --- |
|  |  | SNPs | I^2^ |
| Loneliness | BMI | 13 | 0.51 |
| Loneliness | Body fat | 13 | 0.51 |
| Loneliness | Triglycerides | 12 | 0.51 |
| Loneliness | CAD | 15 | 0.35 |
| BMI | Loneliness | 68 | 0.94 |
| Body fat | Loneliness | 10 | 0.57 |
| Triglycerides | Loneliness | 54 | 0.98 |
| CAD | Loneliness | 38 | 0.95 |

I^2^ quantifies heterogeneity between the genetic variants in an instrument and indicates whether the ’NO Measurement Error’ (NOME) assumption has been violated. If I^2^ is smaller than 0.9, the NOME assumption is likely to be violated.

*Supplementary Table 10: Cochran's heterogeneity statistic for Inverse Variance Weighted (IVW) bidirectional two-sample Mendelian randomization analyses*

| Exposure | Outcome | *n* | Cochran’s Q | |
| --- | --- | --- | --- | --- |
|  |  | SNPs | Q | *p* |
| Loneliness | BMI | 13 | 89.19 | 7.08E-14 |
| Loneliness | Body fat | 13 | 34.26 | 1.04E-05 |
| Loneliness | Triglycerides | 12 | 37.40 | 9.86E-05 |
| Loneliness | CAD | 15 | 21.85 | 0.082 |
| BMI | Loneliness | 68 | 177.07 | 7.05E-12 |
| Body fat | Loneliness | 10 | 24.70 | 0.003 |
| Triglycerides | Loneliness | 54 | 57.81 | 0.302 |
| CAD | Loneliness | 38 | 51.41 | 0.058 |

**
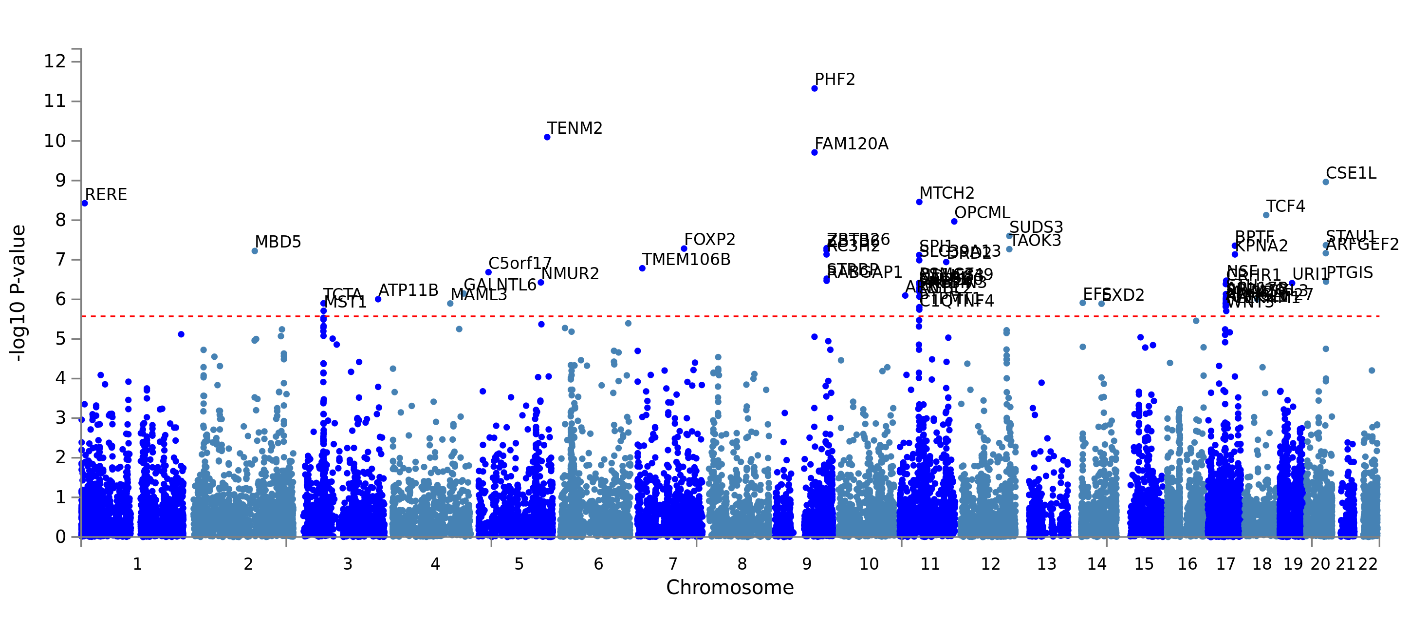
**

Supplementary Figure 1: Manhattan plot of the gene-based analysis showing 38 significantly associated genes

*Supplementary Figure 2. SNP-based heritability (h^2^_SNP_) of lipid values in BioVU and in the discovery dataset (Global Lipid Genetics Consortium). BioVU h^2^_SNP_ values were estimated by restricted maximum likelihood models in GCTA while discovery dataset h^2^_SNP_ values were estimated by LD score regression and extracted from LD Hub (*[*http://ldsc.broadinstitute.org/lookup/*](http://ldsc.broadinstitute.org/lookup/)*).*

*Supplementary Figure 3. Polygenic scores for loneliness are associated with blood levels of HDL and Triglycerides in BioVU. The proportion of variability explained (R^2^) by the loneliness polygenic scores is similar to that of a CAD polygenic score.*

Supplementary Figure 4: Results of the PheWAS on the polygenic score for loneliness, corrected for gender, age, first 10 PCs, and batch. The loneliness polygenic score used here is constructed using only SNPs that reach p<.05 in the GWAS meta-analysis.

***Supplementary Figure 5: Mendelian Randomization results***

Loneliness 🡪 BMI


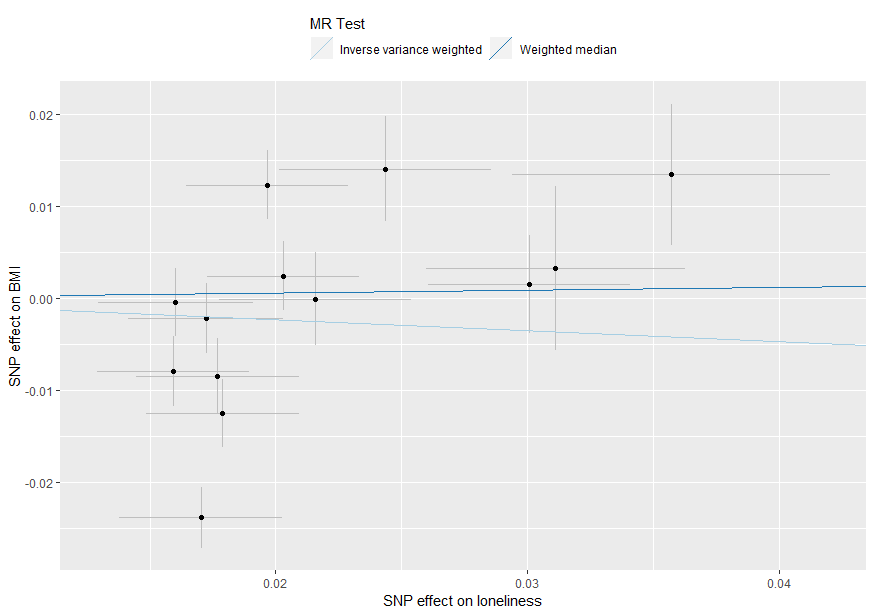


Loneliness 🡪 body fat


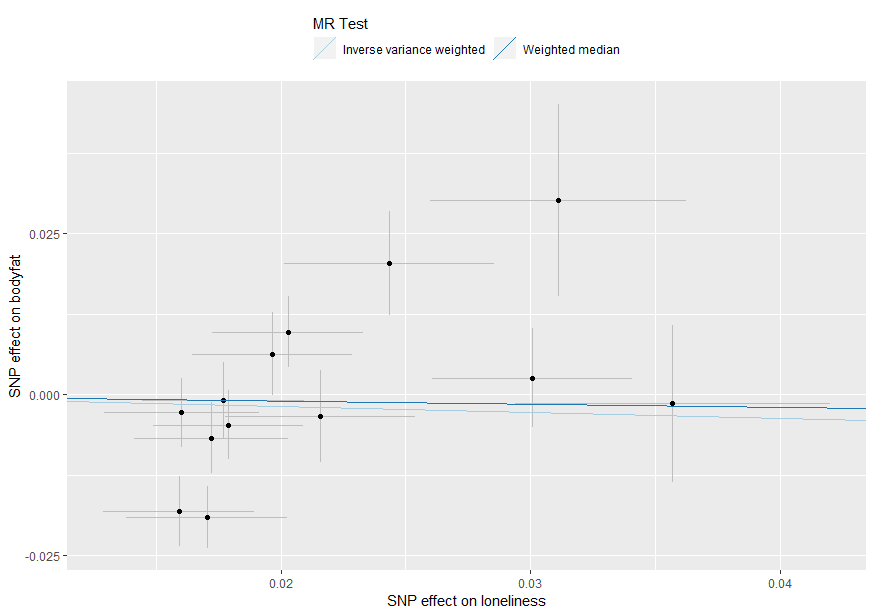


Loneliness 🡪 triglycerides


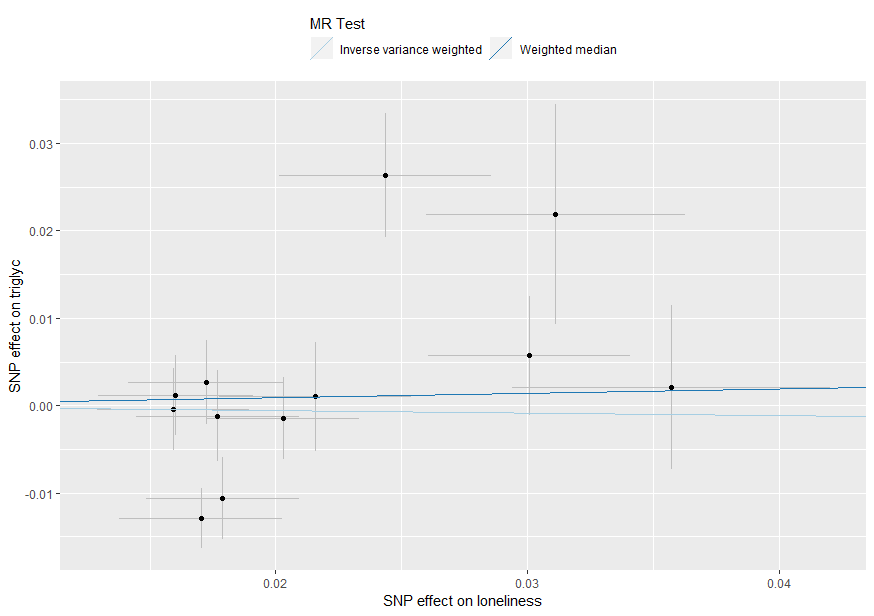


Loneliness 🡪 CAD


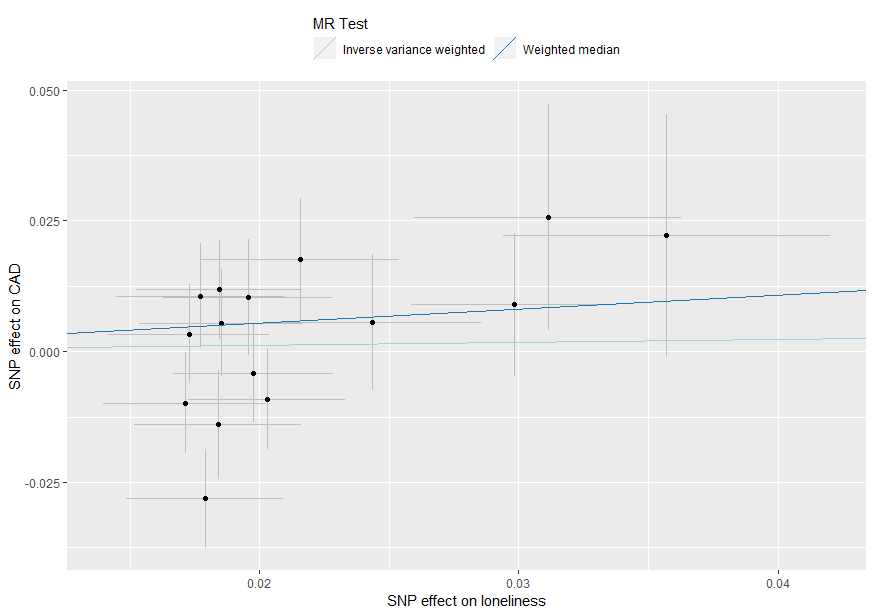


BMI 🡪 loneliness


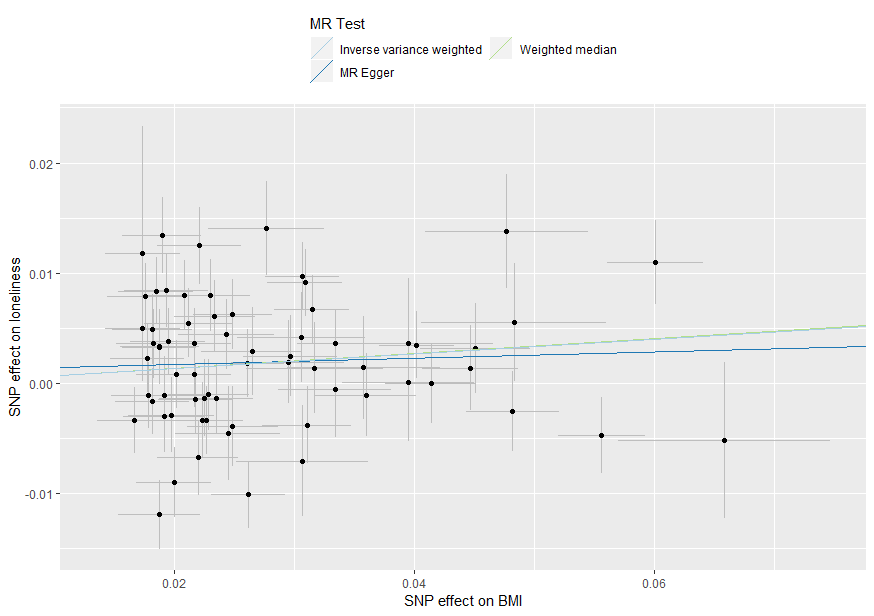


Body fat 🡪 loneliness


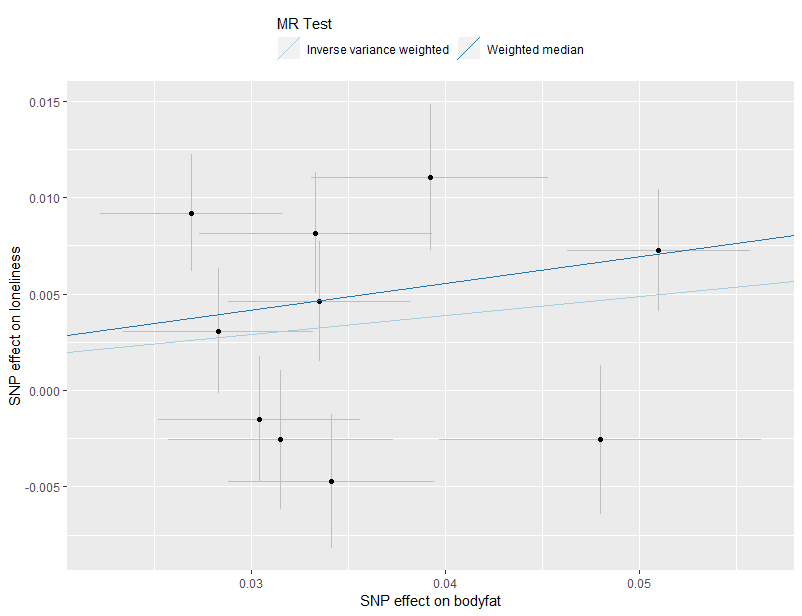


Triglycerides 🡪 loneliness


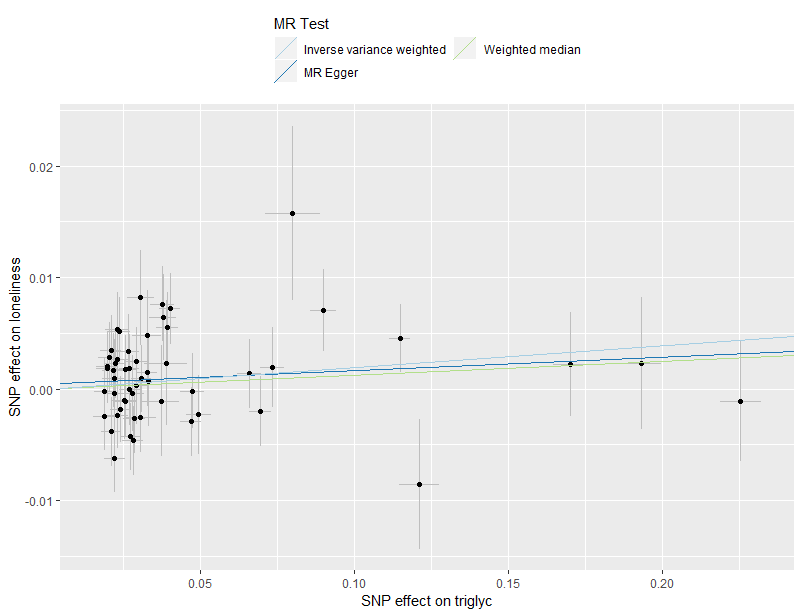


CAD 🡪 loneliness


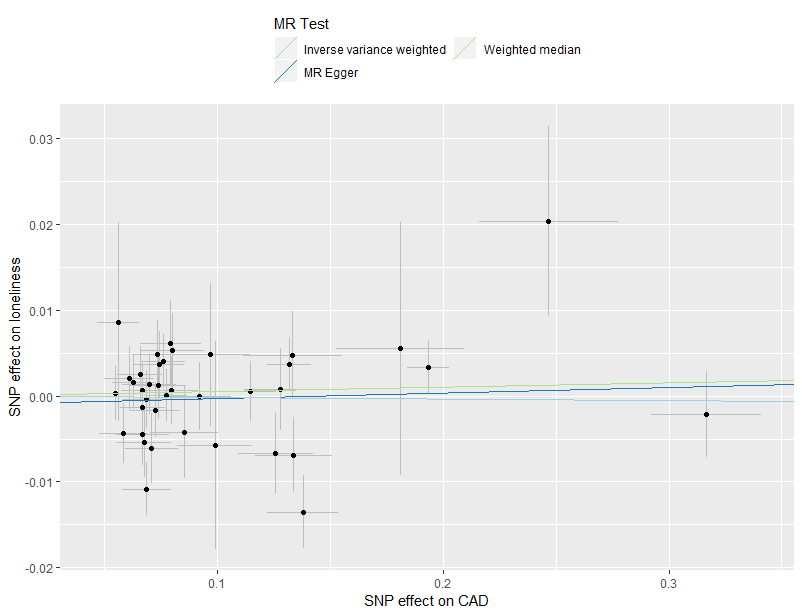

Supplement: SUPPLEMENTARY_loneliness_GWAS_HUM_MOL_GEN_revision_02092019_ref_to_text_ddz219 [file supplementary_loneliness_gwas_hum_mol_gen_revision_02092019_ref_to_text_ddz219.docx]
